# Physiotherapists’ ethical behavior in professional practice: a qualitative study

**DOI:** 10.3389/fmed.2023.1158434

**Published:** 2023-07-17

**Authors:** María Isabel Mármol-López, Elena Marques-Sule, Kati Naamanka, Anna Arnal-Gómez, Sara Cortés-Amador, Ángela Durante, Clara Isabel Tejada-Garrido, Noelia Navas-Echazarreta, Raúl Juárez-Vela, Vicente Gea

**Affiliations:** ^1^Research Group GREIACC, Health Research Institute La Fe (IISLaFe), Nursing School La Fe, Adscript Centre, University of Valencia, Valencia, Spain; ^2^Physiotherapy in Motion, Department of Physiotherapy, Multispeciality Research Group (PTinMOTION), University of Valencia, Valencia, Spain; ^3^Department of Nursing Science/Turku University of Applied Sciences, University of Turku, Turku, Finland; ^4^Department of Nursing, GRUPAC, Universidad de La Rioja, La Rioja, Spain; ^5^Comprehensive Health Care Research Group (INCUiSA) Biomedical Research Center of La Rioja, La Rioja, Spain; ^6^Faculty of Health Sciences, Research Group Community Health and Care (SALCOM), Valencia International University, Valencia, Spain

**Keywords:** professional ethics, physiotherapy, ethics, behavior, qualitative

## Abstract

**Background:**

In health professions, ethics is considered a fundamental competence. The increase in clinical autonomy in the field of physiotherapy is associated with an increase in ethical situations in their clinical practice.

**Objective:**

To explore the ethics of the clinical relationship between physiotherapists and patients, the ethics training received by physiotherapists, and if in the clinical context, physiotherapists identify the necessary attitudes and apply the ethical recommendations of the profession for the ethical situations they experience.

**Methods:**

A qualitative exploratory and descriptive study was performed with physiotherapists. Data were collected through semi-structured interviews. The data were analyzed using content analysis, as proposed by Krippendorf. The study protocol was approved by the University of Valencia Ethics Committee of Human Research.

**Results:**

This study included 15 physiotherapists (66.66% women, average age = 42.2 years), which was sufficient to reach data saturation. We identified four categories: (i) Ethics of the clinical relationship (ethical values, principles, and norms; type of clinical relationship), (ii) Ethics training received (during the physiotherapy studies; current training of students; low importance of ethics in the curriculum), (iii) Necessary attitudes for professional ethical practice (main attitudes were identified: personal attitudes and professional attitudes); (iv) Experiences from professional practice (general; public sector vs. private sector).

**Conclusion:**

The ethics of the clinical relationship between physiotherapists and patients is determined by the attitudes of the practitioner, which are the result of his or her values and previous experiences; and are very centered on ethics of indication (founded mainly on the principles of Beneficence and Non-Maleficence). It is necessary to improve the ethical training received by physiotherapists, which is poorly focused on professional attitudes.

## Introduction

Physiotherapists in their professional practice, as autonomous agents, face complex ethical situations (1), such as understanding and balancing the needs of patients (2), of the patients’ families or other professionals. Other situations such as resource limitations, length, and quality of treatments, as well as billing and working within the constraints and opportunities afforded by health policies and institutional systems, may also occur in daily practice.

The difficulty in making decisions is that they involve situations in which analysis of the facts, questions, and decisions must be based on professional ethical principles. Faced with such questions, physiotherapists must abandon their instinctive or intuitive responses and submit them to an ethical analysis within a clear theoretical framework, without losing sight of the fact that there is not always a single solution to a conflict of these characteristics. These are situations that require deliberation on how to respond and how to integrate ethical principles and professional guidelines. To become a professional, one must learn not only to think in certain ways but also to perform skills, and to practice or act consistently about the norms, values, and conventions of the profession (3–7).

The increasing diversity and complexity of ethical issues and dilemmas which physiotherapists encounter have been reflected in the recent years on formal codifications and guidelines for professional morality: in Spain Professional Ethics in physiotherapy is based on the Code of Ethics principles elaborated by the World Physiotherapy (8), of which the Spanish Association of Physiotherapists is part. There is no consensus on the effectiveness of these principles in guiding a professional (9) and they are problematic concerning their interpretation, multiplicity, and legislation (10). It has been stated that professional codes of ethics are not easily applicable to ethical decision-making when facing an ethical problem (11).

The interest in physiotherapy Professional Ethics has also been reflected in the increased number of articles on the subject. However, Professional Ethics cannot be relegated solely to compliance with deontological codes, but also deals with reflecting on what is the legitimate good of the profession and how to resolve ethical conflicts that arise in practice (2, 5, 12, 13). Several studies have evaluated the inclusion of ethical topics in the curricula of physiotherapy programs and have concluded that ethics is generally included in them. They describe relevant ethical principles and explain frameworks and models for decision-making when facing an ethical conflict during clinical practice (2, 5, 12, 13). However, there are no studies that evaluate the effectiveness of such training, and it is unknown whether this training is subsequently applied during professional practice. In this sense, it seems logical to assert that since ethics is a core competency of physiotherapy practice, it should also be a central component of physiotherapy curricula. Therefore, there is no consensus on the best way to teach ethics in physiotherapy or the appropriate academic load, which is why the amount of taught ethics is highly variable (14). Challenges consist of teaching methods focused on classroom lectures and lack of integration with clinical practice, unqualified ethics teachers, lack of time, and little continuity in the curricula (15, 16).

Research shows that despite the noticeably increased interest in Professional Ethics in physiotherapy, knowledge about this area is still limited and physiotherapists rarely use ethical knowledge and skills to analyze ethical issues raised in their daily clinical practice (13, 17–19). To offer quality physiotherapy care, professionals must identify and consider relevant ethical issues, but it is also necessary that their decision-making is based on the ethical principles of the profession.

The main purpose of this study was to explore the ethics of the clinical relationship between physiotherapists and patients, as well as the ethics training received by physiotherapists. Secondly, we aimed at evaluating if physiotherapists identified in the clinical context the necessary attitudes and experiences related to Professional Ethics.

## Methods

### Design and sampling

The methodological approach was qualitative and exploratory. The study was performed in 2019 at the University of Valencia (Spain).

Given the qualitative and exploratory nature and the objective of the study, a convenience sampling was carried out, and in addition, the participants were asked to nominate colleagues who met the same inclusion criteria (i.e., snowball sampling) (20), who were then contacted to take part in further semi-structured interviews. For sampling, therefore, a combination of convenience sampling with the choice of participants and subsequent snowball sampling, until data saturation was reached, was chosen. Twenty-five professionals were invited to participate in the study, of which 40% declined or did not attend the interview.

A homogeneity and heterogeneity analysis (21) were carried out on the selected sample to design a structural sample that could allow knowing, analyzing, and interpreting different perspectives until obtaining a deep understanding of the study topic. A previous definition of a characteristic profile allowed us to consider a professional physiotherapist as an “expert.” This profile was established from a literature review and the research team’s experience on the subject, thus participants presented an “expert profile” including criteria considered as quality indicators that covered different aspects of the profession. Therefore, it was considered as an inclusion criterion having a minimum experience in 3 of the 4 following professional functions: (i) Clinical practice: 5 or more years of clinical practice; (ii) Teaching: undergraduate university teaching (minimum 1 year) or postgraduate teaching (master’s or specialization); (iii) Research: scientific publications and/or attendance to scientific congresses; (iv) Management: clinical or academic management position.

For recruitment, two members of the research team screened potential participants based on the study inclusion criteria, and contacted them by email, phone, or in person.

In this interview, the characteristics of the study were widely reported, obtaining their verbal acceptance. Subsequently, they were emailed information sheets for study participation and acceptance for recording the interview. Both written informed consents were completed and signed at the time of the in-person interview.

### Information gathering procedure

The data were collected via semi-structured interviews conducted by two of the team’s researchers, between March and October 2019. The semi-structured interviews were carried out in a private, quiet, and isolated environment, and through focused individual, in-depth interviews with a flexible script based on the research questions. All the interviews were conducted in Spanish. The content of the interview was developed by two university professors who were experts in Professional Ethics and Physiotherapy. The questions were designed solely for this study and were based on both their experience of working as a physiotherapist and teaching Professional Ethics. The proposed questions were further discussed with two nursing university professors and experts in qualitative analysis. For data collection, in addition to video recording (informed consent was previously obtained), a data collection notebook was designed to record the appropriate notes by the interviewer (22).

Each interview lasted around 90 min and was videotaped for later analysis of its content. The number of semi-structured interviews was determined by data saturation (i.e., when no new findings or concepts emerged from the interviews) (22). Initially, a short presentation of about 3 min was made to clearly define the topic and objectives of the study, as well as to generate an adequate atmosphere and communicative interaction between interviewer and interviewee. The interviewer adopted a neutral position, in which the interviewee was treated as equal. The order in which the various topics were addressed and the way of asking the questions were left open to the decision and assessment of the interviewer, adapting the questions according to the dynamics of the conversation (23).

After collecting baseline data to frame the sociodemographic and professional profile of the participants, the interview began with certain questions formulated sequentially. Sample questions are shown in Table 1.

**Table 1:** Sample questions from the semi-structured interviews.

1	Regarding ethical principles and behavior, what is the first thing that comes to your mind? Does it refer you to some other concept?
2	What do you consider an ethical behavior at a professional level is?
3	Which attitudes do you consider basic for the professional development of physiotherapy?
4	Do you think that the training acquired in ethics during your physiotherapy degree was enough? Did the clinical training practices help you?
5	What differences in the current ethics training would you highlight compared to when you studied the degree?
6	Do you keep updated on the procedures you perform to ensure the highest quality physiotherapy care?
7	In your experience, how did you acquire the ability to act in situations that imply an ethical dilemma?
8	Have you ever delegate or would you ever delegate your functions as a physiotherapist to other professionals in order to make a physiotherapy service more profitable? Why?
9	Would you give a patient a privileged treatment in a certain situation? Why?
10	Does it seem fair to you that immigrants access our health system for free and receive physiotherapy treatments?
11	What would you do if you discovered that a patient is a pretender? Would you continue the treatment or stop it?
12	Have you ever been convinced that a treatment is going to work very well on a patient, and you have applied it without informing her/him, and without asking for her/his consent? Should physiotherapists ask for informed consent?
13	Have you ever treated, or would you treat a patient who suffers from a condition you do not know and are not trained for?
14	Have you identified any ethically questionable situation or that could compromise professional ethics in other colleagues? Could you describe these situations?
15	What do you think of a situation in which your partner refuses to treat a patient because who is of another race? What would you do?
16	How have you felt, or would you feel if you saw a colleague hurt a patient by applying an unnecessary technique?
17	In what circumstances in your life do you think you would be able to act against ethical principles?

As the interviews unfolded, these initial broad questions were outlined until the categories or theoretical concepts that explained the discourses emerged. The categories developed facilitated the understanding of the different scenarios and experiences (23, 24). The interviewer gave the interviewees relative freedom to guarantee the richness of the speech and allow latent information to emerge in the form of categories and subcategories.

### Data analysis

Digital recordings from all semi-structured interviews were transcribed verbatim. Data were analyzed using content analysis, as proposed by Krippendorf (25). A narrative analysis of the content of the recordings and the transcripts of the interviews was carried out. The transcripts were reviewed by listening to the recordings, which ensured the reliability of the data. All interviews were read several times to get an impression of the whole text. The analysis process was carried out in three phases: factual analysis (content), intersubjective (meanings), and symbolic (interpretation) (24, 26). To guarantee the reliability of the process, the coding of each of the transcripts was carried out through triangulation between the members of the research team.

Through careful review and continuous comparison of the data, categories and subcategories were obtained mainly from inductive reasoning, by observing both the manifest and the latent contents (27). The manifest content was obtained from the transcription of the verbal responses of the study participants, which made explicit the difference in categories and subcategories of information. Final categorization and subcategorization were obtained from latent content expressions that made it possible to extract the meaning and attributes of the underlying discourse (28). Only after the categories were identified and confirmed, quotations were translated into English by the researchers.

From the beginning of the investigation, the interviews were coded by the study’s principal researcher (MIML) and by another researcher who assumed the role of assistant (VGC), to ensure the reliability and consistency of the results, and to provide an assessment of the reliability between researchers.

More than 90% of the obtained codes were consistent between both researchers. The results of each interview were discussed among members of the research team and content revisions were conducted when necessary. Following content reviews, the codes were stable. The principal investigator was also involved in the entire process, to ensure consistency and adequacy of the process. Finally, as a quality criterion, the COREQ 32-item Checklist was followed to guarantee reliability (29). Several strategies were used to ensure the credibility and trustworthiness of the data, including multiple research team members reviewing the transcriptions (MIML, VGC, EMS), multiple team discussions to identify categories, and member checking and coding verification by a second team member (VGC).

### Ethical considerations

Informed consent was obtained in July 2018 by the Ethics and Research Committee of the University of Valencia (H1531842325974). Informed consent documents were designed for study participation and the treatment of the image and sound of the study participants after recording, as well as a study-specific confidentiality commitment. The interviews were stored on digital equipment with a secure password and respecting European regulations and protection data regulations in Spain (Personal Data Protection Act (LOPD) 15/1999, December 13). An effective anonymization system was established that did not allow the subsequent identification of the study subjects.

All participants were informed of the objectives of the project and voluntarily signed their consent to participate in it, being able to revoke it if they chose. To guarantee compliance with Organic Law 1/1982, of May 5, on civil protection of the right to honor, personal and family privacy, and one’s image, the express consent of the participants was asked concerning the use of the images of their voice recordings.

## Results

The interviewees felt satisfied and grateful that someone was interested in their experience and the ethical aspects of their profession.

### Sociodemographic characteristics of the participants

Fifteen physiotherapists participated in the study (average age = 42.2 years, mean time of professional practice = 19.4 years). The homogeneity analysis highlighted that all participants had extensive healthcare practice (5 or more years), performed undergraduate university teaching at the time of data collection, and regularly attended scientific events. 60% of the participants (nine) were working in the private healthcare sector, 40% carried out management activities, 33.3% published research studies, and 26.6% implemented postgraduate teaching (Table 2).

**Table 2:** Sociodemographic characteristics of the participants.

Participants	Age	Gender	Clinical practice experience (years)	Place of work	Undergraduate teaching	Postgraduate teaching	Scientific publications	Attendance to scientific events	Management activities
P1	40	Woman	18	Public hospital	Yes	-	-	Yes	-
P2	47	Woman	25	Public hospital	Yes	-	-	Yes	-
P3	64	Man	38	Public hospital	Yes	Yes	-	Yes	Yes
P4	36	Woman	16	University/Private clinic	Yes	-	-	Yes	Yes
P5	40	Man	18	University/Private clinic	Yes	-	Yes	Yes	Yes
P6	45	Woman	23	Public hospital/University	Yes	-	Yes	Yes	-
P7	27	Woman	5	University/Private clinic	Yes	-	-	Yes	-
P8	37	Woman	16	University/Private clinic	Yes	-	-	Yes	Yes
P9	41	Woman	20	University/Private clinic	Yes	-	-	Yes	-
P10	64	Man	36	Public hospital/University	Yes	Yes	Yes	Yes	Yes
P11	30	Woman	8	University	Yes	-	Yes	Yes	-
P12	36	Woman	16	Public hospital/Private clinic	Yes	-	-	Yes	-
P13	41	Man	16	Public hospital/Private clinic	Yes	-	-	Yes	-

### Participants’ discourse on professional ethics

**Figure 1:**
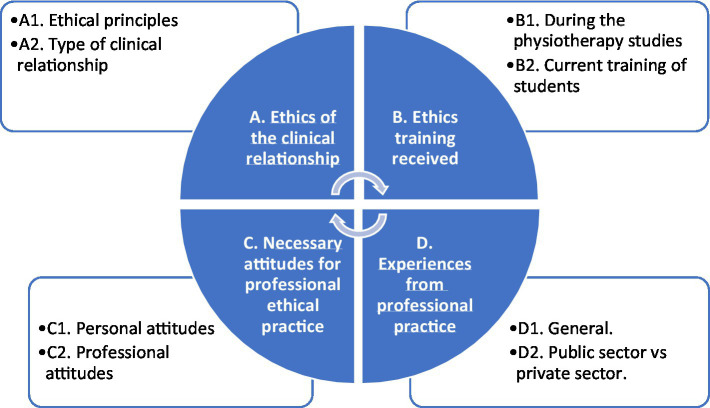
Categories and subcategories on ethical aspects in physiotherapy professionals.

- From the inductive analysis of the data, four main categories emerged, Ethics of the clinical relationship (A), Ethics training received (B), Necessary attitudes for professional ethical practice (C), and Experiences from professional practice (D) (Figure 1). Each category included two subcategories (A1, A2, B1, B2, C1, C2, D1 and D2). In what follows, key findings are described, and illustrated by data excerpts.

Described below through passages from the interviews:

#### A: ethics of the clinical relationship

This category explored the ethics that underlie the clinical relationship, differentiating the ethical principles (A1) and the type of clinical relationship (A2).

##### A1: ethical principles

All participants highlighted the importance of carrying out ethics based on values, from which respect for the rights and dignity of the patient is considered a free and autonomous subject. Other principles that must be present in the clinical relationship are professional responsibility, equity, justice, humanization, commitment, self-determination, confidentiality, privacy, and information to the patient. Some participants observed:

*P1. “… patients have to be treated with good manners and respect.”*

*P5. “… self-determination, respect, autonomy,” “…it is the patient’s freedom.”*

*P6. “… respect, dignity of the patient,” “…confidentiality,” “…autonomy.”*

*P13. “… autonomy, respect and humanized attention.”*

##### A2: type of clinical relationship

Some interviewees stated that in the clinical relationship, a climate of trust should be established to create empathy, personalization, and a helping relationship. The factors that have been highlighted to intervene in the clinical relationship are the cultural level and attitude of patients and physiotherapists. Other factors such as high healthcare pressure, little time for patient care, technology, or bureaucratization of care, sometimes make it difficult to establish a correct professional relationship and therefore depersonalize the relationship. Several participants talked about such relationships:

P4. “…you have to empathize but keep your distance at the same time, some patients step over their role,” “…too many patients are treated at the same time.”

*P9. “…the type of relationship that is established is a helping relationship.”*

*P10. “…the system itself creates problems in the clinical relationship … many patients, standardization of the time for treatment, dehumanization of care ….”*

*P13. “…establishing a climate of trust and a helping relationship is very important.”*

#### B: ethics training received

In this category, the ethical training that the interviewees received (B1) and the training that physiotherapy students currently receive (B2) were compared.

##### B1: during your physiotherapy studies

All participants stated that they had not received ethical training during the physiotherapy studies. This was given in a transversal way and the competence was acquired through the attitude of the teachers and the professionals who supervised the practices. Several participants reported the following information:

*P1. “… there was something related to law, but I do not remember a specific ethics subject,” “… the situations and possible ethical dilemmas that might appear were solved in the practices.”*

*P3. “… I have learned through clinical practice with unexpected situations that I have encountered.”*

P4. “… I received the knowledge of legislation but not of ethics.”

*P6. “… I did not receive specific training in my career, I learned from teachers and professionals.”*

##### B2: current training of students

All the participants agreed that there is currently more training in ethics than in the past, but it is not sufficient because basic individual education is also lacking. Current training includes important aspects that were not included in previous curricula, such as, for example, continuity of care, the relationship of trust, the development of the human dimension, or evidence-based clinical practice…

*P3. “… before we treated pathologies, how we treat people,” “…most of the students are vocational and establish a good relationship with the patient.”*

*P4. “…now the contents are more consistent with the profession … ethics is being applied, and students are better trained.”*

*P6. “…it is difficult for them to see the importance of ethics, and this is shown in some situations in which private conversations are held in front of patients, other patients are discussed, or the use of mobile phones in practices.”*

#### C: identification of the necessary attitudes for professional ethical practice in physiotherapy

In this category, the attitudes that participants considered necessary for professional practice were identified, differentiating between personal attitudes (C1) from professional attitudes (C2).

##### C1: personal attitudes

All participants highlighted the importance of beliefs and values in personal attitudes. The basic attitudes that participants identified in the physiotherapists were to be communicative, helpful, close, empathetic, respectful, sensitive, responsible, assertive, committed, and humanized. Several examples are reported as follows:

*P1. “… communicative attitude is very important … empathy and assertiveness.”*

*P4. “… the affective component is very important, offering closeness and the attitude of helping others.”*

P9. “… commitment and responsibility are necessary ….”

*P14. “… you have to show empathy and sensitivity.”*

##### C2: professional attitudes

Most of the interviewees stated that the necessary professional attitudes for ethical behavior are: having autonomy in professional practice based on knowledge and experience, informing the patient through signing the informed consent, guaranteeing the autonomy of the patient, considering the opinion of the patient, achieving a climate of trust and respect based on active communication, favoring a safe environment with privacy and confidentiality, minimizing pain and establishing a therapeutic attitude of help. All of them are attitudes necessary for the humanization and personalization of the physiotherapist’s care. Participants described examples of professional attitudes such as:

*P2. “…act as best as possible using the best techniques for the patient’s recovery,” “…the patient has to be informed.”*

*P3. “…do what I have to do, respecting the patient, without judging,” “… patient’s safety is a priority at all times,” “… we must minimize pain but make them understand that pain is not a whim.”*

P6. “…a behavior that is within what is correct, respecting not to produce pain,” “… inform and facilitate the informed consent “, “… good therapeutic attitude toward the patient.”

*P8. “…apply what the patient needs and not according to the physiotherapist,” “… we must have the patient’s opinion and establish a therapeutic relationship … informed consent is necessary but is rarely written.”*

#### D: experiences from professional practice

This category showed some situations experienced by the participants in their professional practice and ethical situations in which healthcare ethics were compromised or could be compromised. This category was classified into two subcategories: general experiences (D1) and experiences depending on working in the public and/or private healthcare environment (D2).

##### D1: general experiences

Most of the interviewees stated that they had not experienced any discriminatory experience regarding patients about race, culture, or immigration. 6 participants highlighted that ethics decreased due to unnecessary harm, 5 pointed out the attention to the patients who simulated symptoms or problems compared to the rest of the patients, 4 the possible favor in treatment to certain patients, 2 the violation of privacy and intimacy, 2 declared having suffered reverse discrimination of the patients due to the fact of being a woman, 1 reported the application of rigid treatment protocols that depersonalized care and 1 stated that they had witnessed unethical situations due to political ideology. Some participants commented on their experiences:

*P3. “…sometimes a favor in treatment can be thought of if it is justified but never undermining the rights of other patients,” “… the privacy and intimacy of the patient must be guaranteed.”*

*P7. “…I have seen colleagues causing unnecessary harm but I avoid conflicts, I do not dare to say anything,” “… I have not seen discrimination based on immigration, but health tourism does not seem right to me … I have suffered positive discrimination for being a woman from some of my colleagues.”*

*P8. “…an unethical situation is the one that occurs when the protocol does not allow administering a treatment to a patient although knowing that it would have good results in that patient … our decisions are not ethical for reasons beyond our control … sometimes there are very strict treatment protocols that leave little room for personalized treatment.”*

*P10. “…quantifying unnecessary harm is complicated, how can it be done?,” “… care for the simulator patient is legally complicated, it should be treated like any other patient,” “… when favor in treatment is done, a dichotomy arises, doing more or less it is very complicated,” “… there continues to be paternalism and gender issues.”*

##### D2: public sector vs. private sector

Half of the interviewees expressed there are differences in care in the public and private sectors, such as care for the patient who simulated symptoms or problems, the types of treatment applied highlighting manual therapy, economic criteria, favorable treatment to certain patients, and the type and time of application of treatment. Many participants commented on the differences, by giving examples such as:

*P5. “… different treatments are depending on whether it is in a public or private center,” “in the public sector, the patient is not touched and the patient wants to be touched,” “… sometimes the principle of autonomy is not respected,” “… there are inappropriate treatments, insufficient and even excessive for different reasons such as when they come from insurance companies,” “… sometimes the patient is forgotten and only what needs to be done is done,” “… the rehabilitation doctor does not count on the physiotherapist.”*

*P8. “…in the private sector there are sometimes conflicts of payment with the treatments, what you can charge and what you cannot,” “… the least ethical situations that arise are those derived from private insurance companies due to economic issues.”*

*P11. “…in the public sphere there are more unethical situations due to the short time of treatment…informed consent is never requested.”*

*P14. “…the treatments are the same in public and private sectors, what varies is the duration of the sessions for example, and the type of treatment.”*

**Figure 2:**
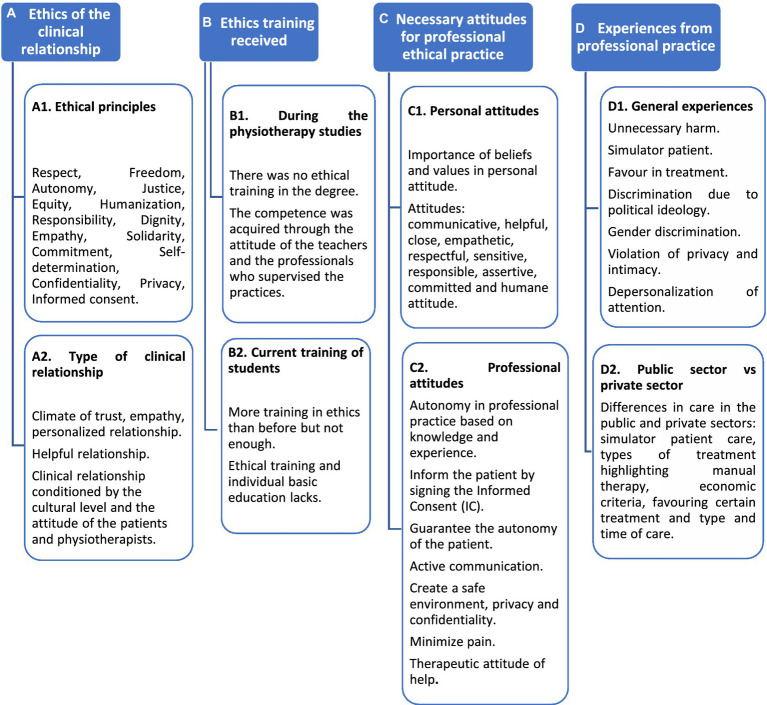
Discourse on the knowledge and ethical application of physiotherapists.

Information units of the categories and subcategories A, B, C, and D are summarized and shown in Figure 2.

## Discussion

This study revealed some aspects regarding the clinical relationship, in terms of the ethical principles and type of relationship, underlining also the necessary personal and professional attitudes of the physiotherapist. Regarding the ethical training and professional attitudes received by physiotherapy students, there is low homogeneity in the curricula, and, in general, teachers are not sufficiently qualified; and students do not perceive it as important within the curriculum. As a strength, this is the first study that has explored the ethical behavior of physiotherapists in their professional practice, and the insights gained from this study will contribute to the body of knowledge on Professional Ethics in Physiotherapy.

According to previous research, physiotherapists do not usually refer to ethical principles or guidelines in ethical decision-making (30, 31). These guidelines should be specific enough in different professional fields, but still loose enough to be applied to different situations and leave room for the professional’s thinking.

Ethical principles should help in addressing ethical issues by helping to identify an ethical issue and justifying why a certain action ought to be preferred over another. According to Meine and Dunn (9), codes that are goal-oriented and include operational guidelines would better support the professional in ethical decision-making. In a previous study, Naamanka et al. (31) stated, that the principles the physiotherapists mostly emphasized were a respectful attitude toward patients, honesty, justice, equality, self-determination, humanity, and professionalism including a professional attitude toward a patient but also the expertise and professional skills. This study complements these findings by also addressing confidentiality and privacy issues. The patient’s active role in the success of the treatment and in decision-making (32) challenges physiotherapists to be ethically sensitive and communicative, to listen and notice what is important for the patient, and his/her attitude also promotes the patient’s engagement in physiotherapy (33).

The results highlighted the type of clinical relationship, in terms of empathy, personalization, and a helping relation, while constraining issues were short time for patient care, and employers putting pressure on the physiotherapists. Ethical problems in a situation when the patient does not receive the amount of physiotherapy that is needed, for whatever reason, often due to resource issues (34) and the pressure on the physiotherapist are experienced as distress (35). A close physical and emotional relationship between the patient and the physiotherapist also creates specific ethical issues (36), such as how to keep a professional distance, for example in a situation when a patient visits the same physiotherapist regularly and for a long time.

As this study pointed out, there is currently more ethics education available than before. Still, ethics education during healthcare professionals’ studies seems to be poorly integrated into the respective curricula, and after graduating, physiotherapists rarely take part in further ethics education (19). Ethics is taught in a non-standardized way (37), there is a shortage in clinical ethics education, and qualified staff to teach ethics is lacking (15). This raises concerns because of the evidence that underlines that one’s ethical competence has an impact on the professional’s strength and awareness to engage in ethical processes at the workplace, which is associated with optimal solutions for the patients and decreased moral distress of the staff (3). As the participants in our study seem to suggest, increasing teaching about ethical theories and frameworks, as well as clinical ethics practice in physiotherapy programs could facilitate physiotherapy students’ and professionals’ understanding of what is an ethical issue and how such issues can be resolved or dealt with (38). These curricular aspects are confirmed by the results of our study in Spain; even though the Spanish physiotherapy curricula include theoretical and practical training in professional legislation and ethics, we have seen how most of the participants recognize that the training could be improved and more oriented to professional dilemmas and practice. Therefore, one option is to modify the curricular design to achieve a greater number of training credits and content more closely linked to praxis.

The results of the study, concerning the general experience of the ethical situations in their professional practice are mostly in line with a previous study stating, that most of the ethical issues encountered in physiotherapy practice concern providing high-quality care and allocation of resources (financial considerations), equality, self-determination and relationships with patients and other healthcare professionals (39). This study highlights also the lack of privacy issues as well as discrimination due to gender or political viewpoints. The attitudes related to how the therapists prioritize between patients based on their socioeconomical status (40), and the asymmetrical relationship between the therapist and the patient (32), have been found problematic in previous studies.

Previous research has identified a gap in knowledge concerning the impact of the setting on encountering ethical problems. There are studies focused on ethical problems in the public sector (39) as well as in private sector physiotherapy (38). This study revealed some experience-based situations concerning the differences in public and private sector physiotherapy, such as the type of treatment applied in the private sector, economic criteria, favorable treatment to certain patients in the private sector, and treatment duration. According to Hudon et al. (38), economic ethical issues, that are more salient in the private sector, can pose challenges to physiotherapists, such as conflicts of interest, cherry-picking practices, lack of time affecting the quality of care, dual agency, and product sales. In our study, we observed that it is precisely in these cases where honest professional attitudes and ethics based on fundamental principles allow us to make the best decision for the patient.

### Limitations

The study was carried out in one center in Spain, thus any generalizations should be cautioned. Future research is warranted to explore the ethical behavior of physiotherapists in other countries and contexts. Additionally, the behavior of physiotherapy students in their clinical practices might be also studied, and therefore results could be compared, although comparing qualitative data may pose a challenge. Quantitative research about the behavior of physiotherapists in their professional practice could be implemented. Future studies could use mixed methods, comparing results for quantitative and qualitative content.

## Conclusion

The ethics of the clinical relationship between the physiotherapist and the patient is very much determined by the attitudes of the practitioner, which are the result of his or her values and previous experiences. Physiotherapists identified in the clinical context the necessary attitudes, but currently many physiotherapists focus strictly on ethics of indication, founded mainly on the principles of Beneficence and Non-Maleficence. An attitudinal change is therefore essential in terms of ethics, focusing on a model of shared responsibility where the principles of patient autonomy, freedom, judgment must be involved, generating an interaction in which the physiotherapist influences the clarification and determination of the best treatment for the patient while respecting the dignity of the individual. To this end, it is necessary to improve the ethical training received by physiotherapists, which is poorly focused on professional attitudes.

## Data availability statement

The raw data supporting the conclusions of this article will be made available by the authors, without undue reservation.

## Ethics statement

The studies involving human participants were reviewed and approved by Ethics and Research Committee of the University of Valencia (H1531842325974). The patients/participants provided their written informed consent to participate in this study.

## Author contributions

MM-L and EM-S: conceptualization and writing—original draft preparation. KN: methodology. AA-G, ÁD, and SC-A: formal analysis and writing—review and editing. EM-S, SC-A, and AA-G: investigation. RJ-V: resources. VG: data curation. RJ-V and VG: supervision. ÁD: project administration and funding acquisition. All authors contributed to the article and approved the submitted version.

## Conflict of interest

The authors declare that the research was conducted in the absence of any commercial or financial relationships that could be construed as a potential conflict of interest.
